# High Prevalence of HIV Infection and Bisexual Networks among a Sample of Men Who Have Sex with Men in Eastern China

**DOI:** 10.1371/journal.pone.0129300

**Published:** 2015-06-08

**Authors:** Haijiang Lin, Yingying Ding, Xing Liu, Qionghai Wu, Weiwei Shen, Na He

**Affiliations:** 1 Department of Epidemiology, School of Public Health, Fudan University, and The Key Laboratory of Public Health Safety of Ministry of Education, Shanghai, China; 2 Taizhou City Center for Disease Control and Prevention, Taizhou city of Zhejiang Province, China; 3 Collaborative Innovation Center of Social Risks Governance in Health, Fudan University, Shanghai, China; Temple University School of Medicine, UNITED STATES

## Abstract

**Objective:**

To examine homosexual and heterosexual behaviors, behavioral networks and HIV infection among men who have sex with men (MSM) in Eastern China.

**Methods:**

A cross-sectional survey was conducted among MSM in 2013 in a rural prefecture of Zhejiang province. Participants were interviewed for their sexual behaviors and sexual networks and were tested for HIV infection.

**Results:**

A total of 620 MSM from gay bath houses and bars participated in the survey. Of them, 58.2% aged 18 to 39 years and 49.5% were currently married with a female. The age of first homosexual contact was 26.7 years on average, ranging from 12 to 66 years. 91.0% had multiple male sex partners and 86.1% also had female sex partners in lifetime. 70 (11.3%) of the participants were tested HIV-positive. A total of 620 independent egocentric sexual networks involving 620 study participants and 1,971 reported sexual partners in the past 12 months were constructed, including 70 networks for the 70 HIV-positive participants with their 221 sexual partners and 550 networks for the 550 HIV-negative participants with their 1,750 sexual partners. The median network degree was 3 (IQR 2-4) overall and was not different between HIV-positive participants (Median: 3; IQR: 2-4) and HIV-negative participants (Median: 3; IQR: 2-4) (Mann-Whitney test, Z=-0.015, *P*=0.998). The proportion of networks with a multiple male sexual partnership was 63.7% overall, 62.8% for HIV-positive participants and 63.8% for HIV-negative participants (χ^2^=0.025, *P*=0.875). The proportion of networks with both male and female sexual partners was 44.8% overall, 47.1% for HIV-positive participants and 44.5% for HIV-negative participants (χ^2^=0.169, *P*=0.681). Consistent condom use and knowledge of HIV infection status were rare within the network partners.

**Conclusions:**

The currently high HIV prevalence and complicated bisexual networks among MSM in the study area provides enhanced evidence for developing tailored prevention strategies for HIV transmission among and beyond the MSM population.

## Introduction

Human immunodeficiency virus (HIV) is now spreading at an alarming rate with substantial increases in prevalence among men who have sex with men (MSM) in recent years across all regions of China. A national survey of 57,407 MSM in 61 cities in China during 2008–2009 revealed an overall HIV prevalence of 4.9%, varying from over 10% in the south-west, to 7% in the east and 4–5% along coast in the south and north-east[1]. Nationally, the proportion of the newly reported HIV cases infected through homosexual transmission increased from 2.5% in 2006 to 21.4% % in 2013 (Unpublished Data from China Center for Disease Control and Prevention). Among the 780,000 people estimated to be living with HIV in China in 2011, 46.5% were infected through heterosexual transmission whereas 17.4% were infected through homosexual transmission [2].

Owing to traditional Chinese culture and family values, an estimated 17–35% of Chinese MSM are currently married, and more than 70% will eventually be married [3–8], while only 1.5–6% are married to women in western countries[9–11]. Prior studies also found that about 50% to 70% of MSM in China reported having had sex with both male and female sexual partners[8, 11–19]. The concurrent homosexual and heterosexual activities among these people could potentially serve as a bridge for HIV transmission between certain high risk group of MSM and general heterosexual population in China [7, 8, 12–21].

Construction of sexual networks of HIV-infected individuals with their sexual partners offers a unique opportunity for visually monitoring potential threats and patterns of HIV spreading at a population dynamic level instead of the individual level which typically asks for personal independent risk behaviors or exposures to the virus [19, 22–24]. This is particularly relevant for Chinese MSM given the complexity of sexual networks, low rates of condom use and increasing HIV prevalence among this population[7, 11, 19, 21, 25, 26]. Nevertheless, no studies have been specifically designed to examine bisexual networks involving both male and female partners for MSM in Eastern China among whom the HIV epidemic is rapidly spreading. To fill this gap, a cross-sectional study was conducted among MSM in a prefecture area in Eastern China. Knowledge gained from this study would be helpful in understanding HIV transmission patterns amongst MSM, men who have sex with men and women (MSMW), and their partners in the study area.

## Methods

### Study site

This study was conducted in Taizhou prefecture of Zhejiang Province, a coastal region in Eastern China, which has a total of 5.9 million residents (National Bureau of Statistics of China, unpublished data). The first HIV case was reported in 1996. By the end of 2012, 1,171 HIV-infected individuals had been diagnosed and registered with the Chinese National Information System for AIDS Prevention and Control (CNISAPC). Of them, 69.9% were infected via heterosexual contacts, 19.1% infected via homosexual contacts, and 5.8% infected via injection drug use (IDU).

### Study participants and data collection

The study was carried out from January to December in 2013. Eligible participants were men who had previously engaged in anal or oral sex with a man and were at least 18 years old. Participants were recruited from gay bars and bath houses and were informed consent to participate in the survey. A face-to-face paper-pencil questionnaire interview was administered anonymously by a trained public health professional to request information of sociodemographic characteristics and sexual behaviors of the participants. Each participant was also encouraged to provide sexual behavioral information of a maximum of six most familiar male and female sexual partners in the past 12 months, including sexual relationship and condom use between the participant and his reported sexual partners. HIV infection status of reported sexual partners was also requested, if knowledgeable to the participant. Such information was solicited by asking the participant three specifically designed questions for each of his reported sexual partners: “what is your sexual relationship (spouse, regular, commercial, casual or others) with this sexual partner in the past 12 months?”, “how often have you used condoms with this sexual partner in the past 12 months?”, and “what is the current HIV status of this sexual partner?”. Each participant was given 30 Yuan RMB (about US$5) for travel reimbursement.

### Voluntary HIV Counseling and Testing

All participants in the study would receive face-to-face pre-test counseling (for an average of 30 to 45 minutes) conducted by a public health professional. Venus blood was collected by an experienced nurse using sterilized needles and sterile tubes. Each plasma sample was coded by a unique identification number and was screened for HIV antibodies using an enzyme-linked immunosorbent assay (ELISA; Vironostika HIV Uni-Form II plus O ELISA Kit, Biomerieux, Boxtel, Netherland) according to the manufacturer’s instructions. Those who screened HIV-positive had their results confirmed by a Western Blot assay (HIV BLOT 2.2, Genelabs Diagnostic, Science PK, DR, Singapore). All tests were performed by two experienced laboratory technicians without knowledge of the personal identity of the study participants. All participants were offered a post-test counseling according to the national guideline.

### Statistical analysis

All statistical analyses were carried out using the SPSS system for Windows Version 17.0 SPSS Inc., Chicago, IL, USA). Descriptive analyses were conducted to elucidate sociodemographic characteristics and sexual behaviors of the study participants by their HIV infection status. Categorical variables were compared using Chi-squared test or Fisher’s exact test where appropriate. Egocentric sexual networks of the participants were diagramed and categorized according to the type and size or network degree of the networks. Network degree is defined as the number of links incident upon an index participant, i.e., the number of ties that an index participant has. Since the network degree in this study was not in normal distribution according to the Kolmogorov-Smirnov test for normality, the distribution of network degree was described with median and interquartile range (IQR) and was compared between different groups using Mann-Whitney test. All tests were two-tailed and P-value<0.05 was considered to be significant.

### Ethical Considerations

The study was reviewed and approved by the Institutional Review Board (IRB) of Fudan University, Shanghai, China. Informed consent in written form was obtained from each participant involved in the study.

## Results

### Sociodemographic characteristics and HIV infection status

A total of 620 MSM were eligible and enrolled in the study. Of them, 58.2% were aged 18 to 39 years, 49.5% were currently married with a female, 52.9% had high school or college education, and 31.0% were non-local residents, i.e., migrants (without local ‘*hukou*’ in Chinese) (Table 1). Seventy participants (11.3%) were tested HIV-positive. As shown in Table 1, sociodemographic characteristics such as age, marital status, education, and official residency status were not significantly different between HIV-positive and HIV-negative participants. However, MSM and MSMW participants were significantly different by age and education. Compared to MSMW, a higher proportion of MSM were aged less than 30 years(82.6% vs. 29.2%) and had an education level of high school or above(84.9% vs.47.8%).

**Table 1:** Sociodemographic characteristics of study participants (N = 620) in Taizhou prefecture of Eastern China, 2013.

		Total	HIV positive MSM	HIV negative MSM
		(N = 620)	(n_1_ = 70)	(n_2_ = 550)
		No. (%)	No. (%)	No. (%)
***Sociodemographics***				
Age (years) (χ2 = 1.85, *P* = 0.60)				
	18–29	227(36.6)	21(30.0)	206(37.5)
	30–39	134(21.6)	15(21.4)	119(21.6)
	40–49	141(22.8)	19(27.2)	122(22.2)
	50-	118(19.0)	15(21.4)	103(18.7)
Marital status (χ2 = 1.35, *P* = 0.51)				
	Never married	249(40.2)	24(34.3)	225(40.9)
	Currently married with a woman	307(49.5)	37(52.9)	270(49.1)
	Divorced or widowed	64(10.3)	9(12.8)	55(10.0)
Official residency status (‘*hukou*’) (χ2 = 2.13, *P* = 0.14)				
	Local	428(69.0)	43(61.4)	385(70.0)
	Non-local	192(31.0)	27(38.6)	165(30.0)
Education (χ2 = 4.19, *P* = 0.12)				
	Illiterate or primary school	59(9.5)	8(11.4)	51(9.3)
	Middle school	233(37.6)	33(47.2)	200(36.4)
	High school or above	328(52.9)	29(41.4)	299(54.3)
***Sexual behaviors***				
Age at first sex with men (years) **(χ^2^ = 8.38, *P* = 0.04)**				
	<18	44(7.1)	3(4.2)	41(7.5)
	18–29	392(63.2)	37(52.9)	355(64.5)
	30–39	114(18.4)	16(22.9)	98(17.8)
	40-	70(11.3)	14 (20.0)	56(10.2)
Lifetime male sex partners (χ2 = 1.57, *P* = 0.67)				
	1	56(9.0)	4 (5.7)	52(9.4)
	2–5	267(43.1)	29(41.4)	238(43.3)
	6–10	137(22.1)	16(22.9)	121(22.0)
	11-	160(25.8)	21(30.0)	139(25.3)
Lifetime female sexual partners(χ2 = 3.83, *P* = 0.15)				
	0	86(13.9)	5(7.2)	81(14.7)
	1	293(47.2)	39(55.7)	254(46.2)
	2-	241 (38.9)	26(37.1)	215(39.1)
Having multiple male sexual partners in past 12 months (χ2 = 0.02, *P* = 0.88)				
	Yes	395(63.7)	44(62.8)	351(63.8)
	No	225(36.3)	26(37.2)	191(36.2)
Having both male and female sexual partners in past 12 months (χ2 = 0.17, *P* = 0.68)				
	Yes	278(44.8)	33(47.1)	245(44.5)
	No	342(55.2)	37(52.9)	305(55.5)

### Sexual behaviors

The age of first sex with a male was 26.7 years on average and ranged from 12 to 66 years. Nearly two-thirds (63.2%) of the participants had the first male-to-male sex at 18 to 29 years old. HIV-positive participants tended to have significantly higher age at first sex with male, compared to their HIV negative counterparts(χ^2^ = 8.38, P = 0.04) (Table 1). Among the participants, 91.0% reported having two or more male sexual partners in lifetime, i.e., a multiple homosexual partnership; 8.1% had commercial male sexual partners and 37.6% had casual male sexual partners in the past 6 months. The proportion of participants having also had female sexual partners in lifetime (i.e., MSMW participants), was 86.1% overall, 92.8% for HIV-positive participants and 85.3% for HIV-negative participants (Table 1).

### Egocentric sexual networks

**Fig 1:**
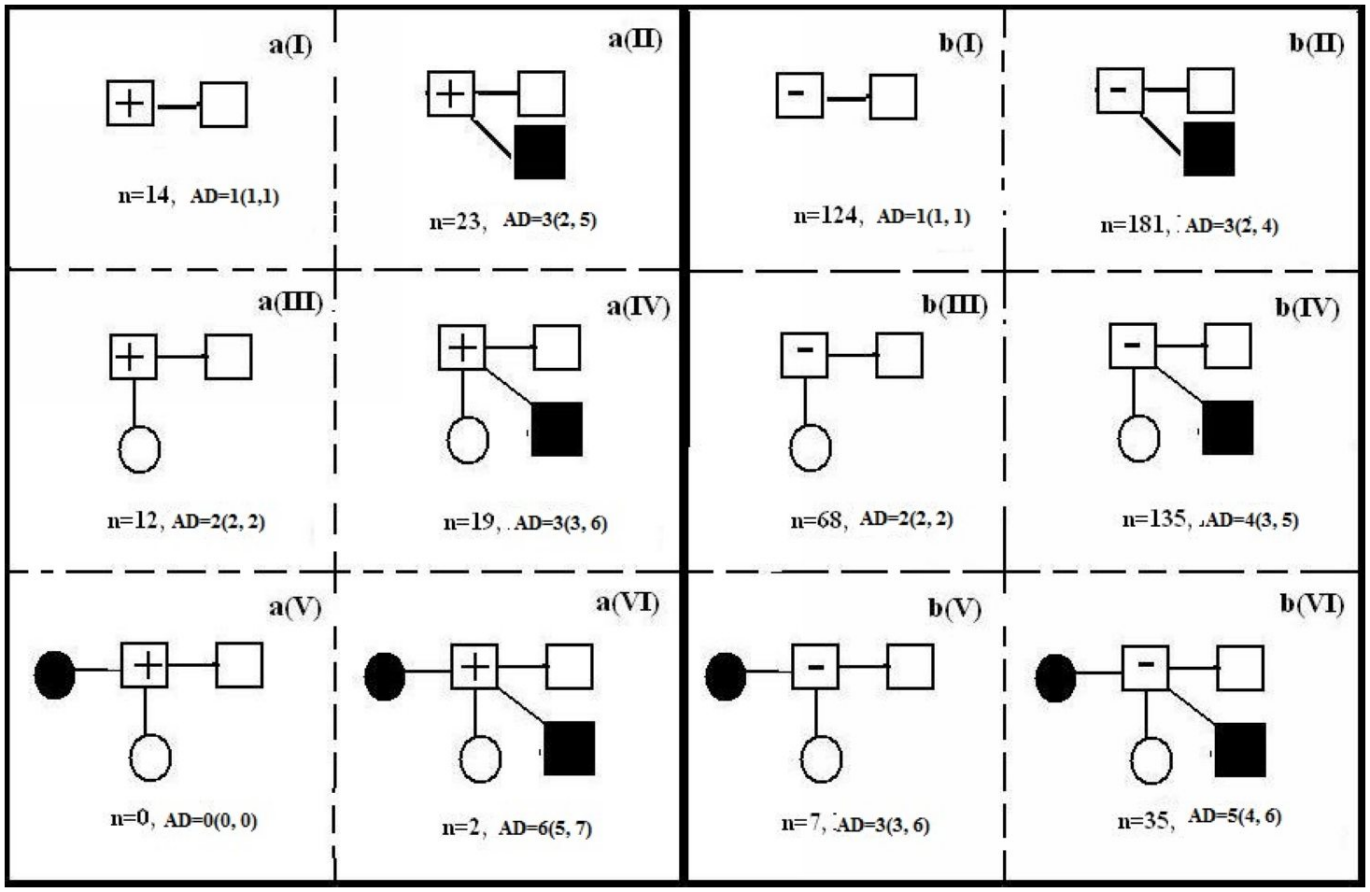
Diagrams and distribution of egocentric sexual networks of HIV-positive (n = 70) vs. HIV-negative (n = 550) participants in the past 12 months. (n: number of sexual networks; MD: the median network degree(Median, IQR).

A total of 620 independent egocentric sexual networks involving 620 study participants and 1,971 reported sexual partners in the past 12 months were constructed, including 70 networks for the 70 HIV-positive participants with their 221 sexual partners and 550 networks for the 550 HIV-negative participants with their 1,750 sexual partners. The median network degree was 3 (IQR: 2–4) overall, 3 (IQR: 2–4) for HIV-positive participants and 3 (IQR: 2–4) for HIV-negative participants, respectively. The network degree has no significantly difference by their HIV status (Z = -0.015, *P* = 0.998). The networks were schematically diagramed and categorized according to the type and size of the networks and were presented in Fig 1. The proportion of networks with a multiple male sexual partnership was 63.7% (395/620) overall, 62.8% (44/70) for HIV-positive participants and 63.8% (351/550) for HIV-negative participants (χ^2^ = 0.025, *P* = 0.875) (Table 2). The proportion of networks with both male and female sexual partners was 44.8% (278/620) overall, 47.1% (33/70) for HIV-positive participants and 44.5% (245/550) for HIV-negative participants (χ^2^ = 0.169, *P* = 0.681) (Table 2).

**Table 2:** Characteristics of egocentric sexual networks in the past 12 months of the survey by HIV status of study index participants, in Taizhou prefecture of Eastern China, 2013.

Sexual behavioral characteristic	HIV positive participants (n1 = 70)							HIV negative participants (n2 = 550)						
Type of egocentric sexual networks*	a(I) (%)	a(II) (%)	a(III) (%)	a(IV) (%)	a(V) (%)	a(VI) (%)	Total (%)	b (I) (%)	b(II) (%)	b(III) (%)	b(IV) (%)	b(V) (%)	b(VI) (%)	Total (%)
**Number of sexual networks**	14 (20.0)	23 (32.9)	12 (17.1)	19 (27.1)	0 (0.0)	2 (2.9)	70 (100.0)	124 (22.5)	181 (32.9)	68 (12.4)	135 (24.5)	7 (1.3)	35 (6.4)	550 (100.0)
**Network degree** ^a^ **Median (IQR)**	1 (1, 1)	3 (2, 5)	2 (2, 2)	3 (3, 6)	0 (0.0)	6 (5, 7)	3 (2, 4)	1 (1, 1)	3 (2, 4)	2 (2, 2)	4 (3, 5)	3 (3, 6)	5 (4, 6)	3 (2, 4)
**Number of sex partners involved in the sexual network** ^b^	14	85	33	87	0	12	221	124	612	132	631	30	221	1750
**Number of male sex partners involved in the sexual network** ^c^	14	85	12	68	_	7	186	124	612	68	496	7	124	1431
**Type of male sex partners**														
Regular	11 (78.6)	19 (22.4)	10 (83.3)	18 (26.5)	_	0 (0.0)	58 (31.2)	95 (76.6)	185 (30.2)	49 (72.1)	136 (27.4)	4 (57.1)	24 (19.4)	493 (34.5)
Commercial	1 (7.1)	3 (3.5)	1 (8.3)	7 (10.3)	_	0 (0.0)	12 (6.5)	4 (3.2)	36 (5.9)	2 (2.9)	31 (6.3)	_	25 (20.2)	98 (6.8)
Casual	2 (14.3)	61 (71.8)	0 (0.0)	43 (63.2)	_	7 (100.0)	113 (60.8)	21 (16.9)	387 (63.2)	14 (20.6)	302 (60.9)	3 (42.9)	69 (55.6)	796 (55.6)
Others	0 (0.0)	2 (2.4)	1 (8.3)	0 (0.0)	_	0 (0.0)	3 (1.6)	4 (3.2)	4 (0.6)	3 (4.4)	27 (5.4)	_	6 (4.8)	44 (3.1)
**Condom use with male sex partners**														
Always	8 (57.1)	30 (35.3)	8 (66.7)	37 (54.4)	_	0 (0.0)	83 (44.6)	51 (41.1)	327 (53.4)	13 (19.1)	220 (44.4)	4 (57.1)	85 (68.5)	700 (48.9)
Sometimes	3 (21.4)	26 (30.6)	3 (25.0)	10 (14.7)	_	5 (71.4)	47 (25.3)	43 (34.9)	178 (29.1)	23 (33.8)	158 (31.9)	1 (14.3)	33 (26.6)	436 (30.5)
Never	3 (21.4)	29 (34.1)	1 (8.3)	21 (30.9)	_	2 (28.6)	56 (30.1)	30 (24.2)	107 (17.5)	32 (47.1)	118 (23.8)	2 (28.6)	6 (4.8)	295 (20.6)
**HIV status of male sexual partners reported by participants**														
HIV+	0 (0.0)	1 (1.2)	0 (0.0)	0 (0.0)	_	0 (0.0)	1 (0.5)	7 (5.6)	6 (0.98)	1 (1.5)	3 (0.6)	_	1 (0.8)	18 (1.3)
HIV-	6 (42.9)	13 (15.3)	5 (41.7)	7 (10.3)	_	0 (0.0)	31 (16.7)	49 (39.5)	175 (28.6)	27 (39.7)	90 (18.1)	1 (14.3)	29 (23.4)	371 (25.9)
Unknown	8 (57.1)	71 (83.5)	7 (58.3)	61 (89.7)	_	7 (100.0)	154 (82.8)	68 (54.8)	431 (70.4)	40 (58.8)	403 (81.3)	6 (85.7)	94 (75.8)	1042 (72.8)
**Number of female sex partners involved in the sexual network** ^d^	_	_	11	19	_	5	35	_	_	64	135	23	97	319
**Type of female sex partners**														
Wife	_	_	10 (90.9)	18 (94.7)	_	2 (40.0)	30 (85.7)	_	_	58 (90.6)	125 (92.6)	7 (30.4)	20 (20.6)	210 (65.8)
Commercial	_	_	0 (0.0)	0 (0.0)	_	1 (20.0)	1 (2.9)	_	_	4 (6.3)	4 (3.0)	1 (4.3)	21 (21.6)	30 (9.4)
Casual	_	_	1 (9.1)	1 (5.3)	_	2 (40.0)	4 (11.4)	_	_	1 (1.6)	6 (4.4)	15 (65.2)	56 (57.7)	78 (24.5)
Others	_	_	0 (0.0)	0 (0.0)	_	0 (0.0)	0 (0.0)	_	_	1 (1.6)	_	_	_	1 (0.3)
**Condom use with female sex partners**														
Always	_	_	0 (0.0)	5 (26.3)	_	1 (20.0)	6 (17.1)	_	_	7 (10.9)	29 (21.5)	10 (43.5)	56 (57.7)	102 (32.0)
Sometimes	_	_	1 (9.1)	2 (10.5)	_	2 (40.0)	5 (14.3)	_	_	12 (18.8)	29 (21.5)	6 (26.1)	23 (23.7)	70 (21.9)
Never	_	_	10 (90.9)	12 (63.2)	_	2 (40.0)	24 (68.6)	_	_	45 (70.3)	77 (57.0)	7 (30.4)	18 (18.6)	147 (46.1)
**HIV status of female sexual partners reported by participants**														
HIV+	_	_	0 (0.0)	0 (0.0)	_	0 (0.0)	0 (0.0)	_	_	2 (3.1)	_	_	_	2 (0.6)
HIV-	_	_	6 (54.5)	9 (47.4)	_	0 (0.0)	15 (42.9)	_	_	26 (40.6)	56 (41.5)	5 (21.7)	32 (33.0)	119 (37.3)
Unknown	_	_	5 (45.5)	10 (52.6)	_	5 (100.0)	20 (57.1)	_	_	36 (56.3)	79 (58.5)	18 (78.3)	65 (67.0)	198 (62.1)

**Note:**

*: a(I) to a(VI), b(I) to (VI) denote the different type of egocentric sexual networks of the MSM participants, details are provided in the Fig 1;

^a, b^: Network degree or the number of sexual partners involved in the sexual network has no significantly difference by the HIV status of the index participants (Z = -0.015, *P* = 0.998)

^c^ Number of male sex partners involved in the sexual network has no significantly difference by the HIV status of the index participants (Z = -0.142, *P* = 0.887)

^d^ Number of female sex partners involved in the sexual network has no significantly difference by the HIV status of the index participants (Z = -0.045, *P* = 0.964).

The proportion of networks with HIV infection status of at least one sexual partner known to the participant was 51.9% (322/620) overall, 44.3% (31/70) for HIV positive participants and 52.9% (291/550) for HIV negative participants (χ^2^ = 1.850, *P* = 0.174). The proportion of networks with HIV infection status of all sexual partners known to the participant was 23.1% (143/620) overall, 22.9% (16/70) for HIV positive participants and 23.1% (127/550) for HIV negative participants (χ^2^ = 0.002, *P* = 0.965). The median percentage of sexual partners with known HIV status within each network was 14.3% (IQR 0–66.7%) among the total of 620 networks, 0 (IQR 0–66.7%) among HIV-positive participants’ networks and 20.0% (IQR 0–66.7%) among HIV-negative participants’ networks (Z = -1.174, *P* = 0.240).

### Partner characteristics of HIV-positive participants

As shown in Table 2, among the 221 reported sexual partners of HIV-positive participants, 186 (84.2%) were male and 35 (15.8%) were female. Of the 186 male sexual partners, 31.2% were regular, 60.8% were casual and 6.5% were commercial. Less than a half of HIV-positive participants (44.6%) reported “always” using condoms with male sexual partners. Most (82.8%) of the 186 male sexual partners had an unknown HIV status, 16.7% were known to be HIV negative and 0.5% were HIV positive. Of the 35 female sexual partners, 85.7% were a wife of the study participant, 11.4% were casual and 2.9% were commercial. Only 17.1% of HIV-positive participants reported “always” using condoms with female sexual partners. More than a half (57.1%) of female sexual partners had an unknown HIV status, 42.9% were known to be HIV negative and none were HIV positive.

### Partner characteristics of HIV-negative participants

Among the 1,750 sexual partners reported by HIV-negative participants, 1,431 (81.8%) were male and 319 (18.2%) were female (Table 2). Of the 1,431 male sexual partners, 34.5% were regular, 55.6% were casual and 6.8% were commercial. About a half (48.9%) of HIV-negative participants reported “always” using condoms with male sexual partners. Most of male sexual partners (72.8%) had an unknown HIV status, 25.9% were known to be HIV negative and 1.3% were HIV positive. Of the 319 female sexual partners, 65.8% were participants’ wives, 24.5% were casual and 9.4% were commercial. The proportion of reported female sexual partners who were a wife of the study participant was significantly lower for HIV-negative participants (65.8%) than that for HIV-positive participants (85.7%; χ^2^ = 5.71, *P* = 0.02). Only 32.0% of HIV-negative participants reported “always” using condoms with female sexual partners. Among the female sexual partners, 62.1% had an unknown HIV status, 37.3% were known to be HIV negative and 0.6% was HIV positive.

## Discussion

MSM is a group with rapidly increasing risk of HIV infection in China[1, 7, 25, 27, 28]. In the present study, the HIV prevalence was 11.3%, much higher than the average national level (7.3%) of HIV infection among MSM of China in 2013 (Unpublished Data from China Center for Disease Control and Prevention), which was very likely attributable to the highly prevalent multiple homosexual partnerships, complex sexual network, casual and commercial homosexual activities of the study participants[19, 26]. The study also revealed that participants having first sex with male at older age were at higher risk for HIV infection, because they tend to be older, and were more likely to have unprotected sex.

Previous studies in China have observed that most MSM also have heterosexual activities[1, 7, 11, 15, 16, 19, 21]. Consistently, 86.1% of the present study participants reported having had lifetime experience of heterosexual contacts and 38.9% reported having had more than one female sex partner. Meanwhile, a half of the participants were married with a woman who accounted for about two-thirds (67.8%) of female sexual partners in the past 12 months reported by the participants. This percentage of being married among the participating MSM was also comparable with that in other areas in China[11, 15]. Ethnographic studies show that Chinese MSM tend to pursue or maintain an open heterosexual or family life while engaging in homosexual activities, mostly due to concerns of extensive stigma and discrimination to homosexuality in Chinese society and important role of a male in carrying on the ancestral line in Chinese culture[20, 21, 29, 30]. Thus, destigmatization to homosexuality in Chinese society is a critical component of sexual health education and promotion programs targeting MSM.

A higher network degree implies more complexity of a sexual network and thus a higher risk of HIV and other sexually transmitted infections[22, 23]. In this study, the median degree of egocentric sexual networks in the past 12 months was 3 and did not vary by HIV infection status of the participants, suggesting that both HIV-positive and HIV-negative MSM in the study area were engaged in complicated multiple sexual partnerships. Nevertheless, consistent condom use was rare within these sexual activities. Moreover, approximately 45% of the networks including nearly a half of HIV-positive participants’ networks involved both male and female partners. These observations suggest the critical bridging role of MSM particularly HIV-infected MSM in HIV transmission across the MSM and the general female populations [21, 31, 32], but more importantly, underscore urgent needs of more effective behavioral intervention programs targeting HIV-infected MSM.

Knowing one’s HIV status is the first step to accessing care and preventing further infection[33–35]. This is particularly relevant for the MSM population in China[19, 26]. However, in the present study, both HIV-positive and HIV-negative participants reported no knowledge of HIV infection status for the most of their most familiar partners especially male sexual or MSM partners. Reasons for such a low knowledge of HIV infection status for sexual partners are unclear but might include concealment or non-disclosure of HIV infection status between sexual partners, low awareness of HIV risks and low access to HIV testing among MSM and their partners. Therefore, identifying MSM and their partners with undiagnosed HIV infection and linking them to medical care and prevention services continues to be a priority for HIV prevention and control in China. Sexual partners in a risky sexual network should be encouraged to receive voluntary HIV counseling and testing, which can be an alternative or supplemental HIV testing strategy for certain high risk yet hard-to-approach individuals.

This study has certain limitations. First, subjects who were recruited from gay bath houses and gar bars might be more sexually active and be at higher risk for HIV. As a result, HIV prevalence and complexity of sexual networks of the study participants might be an overestimation of MSM in Taizhou prefecture. Second, HIV prevalence rather than HIV incidence was measured in the study. It is difficult to determine the magnitude of HIV transmission in this population. Third, the sensitive nature of the questions related to sexual behaviors might lead to information bias due to the stigma or social desirability of certain answers, for example, underreporting the number of sexual partners while overreporting the frequency of condom use. Fourth, data on sexual partners and condom use in the past 12 months prior to the survey were collected, which could lead to recall bias. Finally, egocentric rather than sociometric sexual networks were constructed for participants and characteristic data were collected for a maximum of six most familiar but not all male and female sexual partners of the index MSM participants; future studies should also collect more information on sexual partners to elucidate risk behavioral network characteristics more completely.

Despite these limitations, the results of the study highlight a prevention opportunity that cannot be ignored. The high HIV prevalence and complicated bisexual behaviors among MSM underscore urgent needs of effective intervention programs targeting MSM particularly HIV-infected MSM and their female partners including their wives. The integration of risky behavior and behavioral network analysis provides enhanced evidence for developing tailored prevention strategies for HIV transmission among and beyond the MSM population.
